# Microstructural Insights into Natural Silver Wires

**DOI:** 10.1038/s41598-018-27159-w

**Published:** 2018-06-13

**Authors:** Th. Boellinghaus, V. Lüders, G. Nolze

**Affiliations:** 1 BAM – Federal Institute for Materials Research and Testing, Unter den Eichen 87, D-12205 Berlin, Germany; 2 GFZ – German Research Centre for Geosciences, Telegrafenberg, D-14473 Potsdam, Germany

## Abstract

Due to the increasing global demand for pure silver, native wire silver aggregates in very high purities are gaining more industrial attention. Up to the present, no substantial metallurgical investigation of natural wire silver exists in the accessible literature. To convey urgently needed cross-disciplinary fundamental knowledge for geoscientists and metallurgical engineers, twenty natural wire silver specimens from eight different ore deposits have been investigated in detail for the first time by EBSD (Electron Back Scattering Diffraction), supported by light microscopy and micro-probe analyses. The improved understanding of the natural silver wire microstructure provides additional information regarding the growth of natural silver aggregates in comparison to undesired artificial growth on electronic devices. Clear evidence is provided that natural silver curls and hairs exhibit a polycrystalline face-centered cubic microstructure associated with significant twinning. Although the investigated natural wire silver samples have relatively high purity (Ag > 99.7 wt.-%), they contain a variety of trace elements such as, S, Cu, Mn, Ni, Zn, Co and Bi, As and Sb. Additionally, Vickers micro-hardness measurements are provided for the first time which revealed that natural silver wires and curls are softer than it might be expected from conversion of the general Mohs hardness of 2.7.

## Introduction

For more than 7000 years and already in the early stages of the Neolithic, Chalcolithic and Bronze Age, silver has attracted the interest of mankind as one of the most precious metals. It belongs to the seven elements of the antiquity and very prominent finds of antique silver artefacts have been reported by Heinrich Schliemann during his archaeological excavation of the ca. 5000 years old city of Troja. First commercial silver mining dates back for about 3000 years ago to the mysterious, but metallurgically highly developed Hethitians who lived in a region which now called Anatolia. The Hethitian Empire served for a long historical period as the most prominent source of silver for all ancient western cultures, predominantly for the Egyptians and Greeks. The first smelting and processing technologies are attributed to the same region and started about 2600 years ago when the Chaldeans developed the Cupellation Process to extract silver from silver-rich galena (PbS). They also developed first technologies to subsequently purify the extracted silver further so that it could be processed into ingots, rings, jewelry, dishes as well as ornaments, like for weapons.

While the relevance of silver was mostly related to manufacturing of above mentioned products in the ancient world, silver became increasingly important to the photo industry in the 19^th^ and 20^th^ century. In the modern day industrial use, about 50% of the world-wide annual silver production is used for electronic devices, solar energy, batteries etc. Besides investment requirements about 25% are used in jewellery and silverware manufacturing. Recently, the investigation of silver microstructures has become extremely relevant to the electronic industry, where the instantaneous and unexpected growth of silver whiskers has been associated with respective mal-functions and failures of devices entailing very high safety risks.

Native silver typically occurs in the oxidation zones of silver deposits where Ag-bearing sulfides get oxidized by meteoric water. Thus, silver is mainly processed as major or trace element in many sulfides (e. g. argentite/acanthite, dyskrasite, Ag-sulfosalts, galena etc.) from hydrothermal and epithermal ore deposits but also from shale-hosted mineralization (Kupferschiefer) or from secondary supergene ore deposits. The latter often contain silver enrichments in the form of native silver wires. The growth of native silver is described in detail in the supplementary information. Natural silver can still contain considerable amounts of other elements, as for instance up to 25% mercury in platelets, and should then rather be called an alloy, like amalgam. This means, most of the natural silver has to be processed to higher purities.

However, silver occurs naturally also in much higher purity. Such silver is commonly crystallized in the cubic system and then appears as dendrites, mostly octahedral, but also as cuboidal crystals. As most attractive, not only because of the exceptional high silver grade and easy mining, but also as collectibles have always been regarded specimens with silver grown as hair-like objects on a matrix of acanthite, stromeyerite or other silver-rich ore minerals (cf. Fig. 1 in the SI).

Such type of natural silver appearance is generally described by the term wire silver. It is suggested that wires exhibiting a smaller diameter than 1 mm at the root are also called hair or hair-like silver. For wire silver with larger diameters above 1 mm the expression silver curl appears as more suitable and is thus used here. Silver is known to grow with pronounced longitudinal orientation as single crystal whiskers, based on screw dislocations, as one proposed mechanism. However, natural wire silver exhibits a polycrystalline microstructure and it has still to be clarified, if the growth mechanisms might differ from those of whiskers.

Microstructural investigations of natural wire silver started with some rudimentary studies on the growth of natural silver curls in the early 20^th^ century^1^, but detailed information about such natural silver wires has not been provided^2^. Up to now, only a very rough investigation of the cross section of a single natural silver curl from Freiberg, Germany, is accessible^3^. In contrast, the microstructure of relatively pure artificial alloys for coins and ancient artefacts with silver contents above 95% has recently been investigated^4^. Also, the microstructure of new quaternary silver alloys with good ductility and considerably high strength developed for the jewelery industry has been extensively studied^5–8^. In addition, the metallurgy of artificial silver alloys has been widely evaluated for the electronic industry, due to its exceptional electro-physical properties, especially regarding gold coated specimens^9^.

The present contribution is targeted to bridge nearly hundred years of knowledge gaps in the metallurgy of natural silver curls by utilizing Electron Backscatter Diffraction (EBSD) studies in conjunction with conventional light microscopy to characterize 20 natural silver wire specimens from various localities. They are listed in Table 1 of the SI.

## Results and Discussion

The chemical composition of 17 out of the 20 silver wire specimens has been measured using Electron Microprobe (EMPA). All analyzed samples exhibited a high purity above 99.7 wt.-% Ag, as also described by Kotkova et al^10^. No alloying elements have been found.

**Figure 1:**
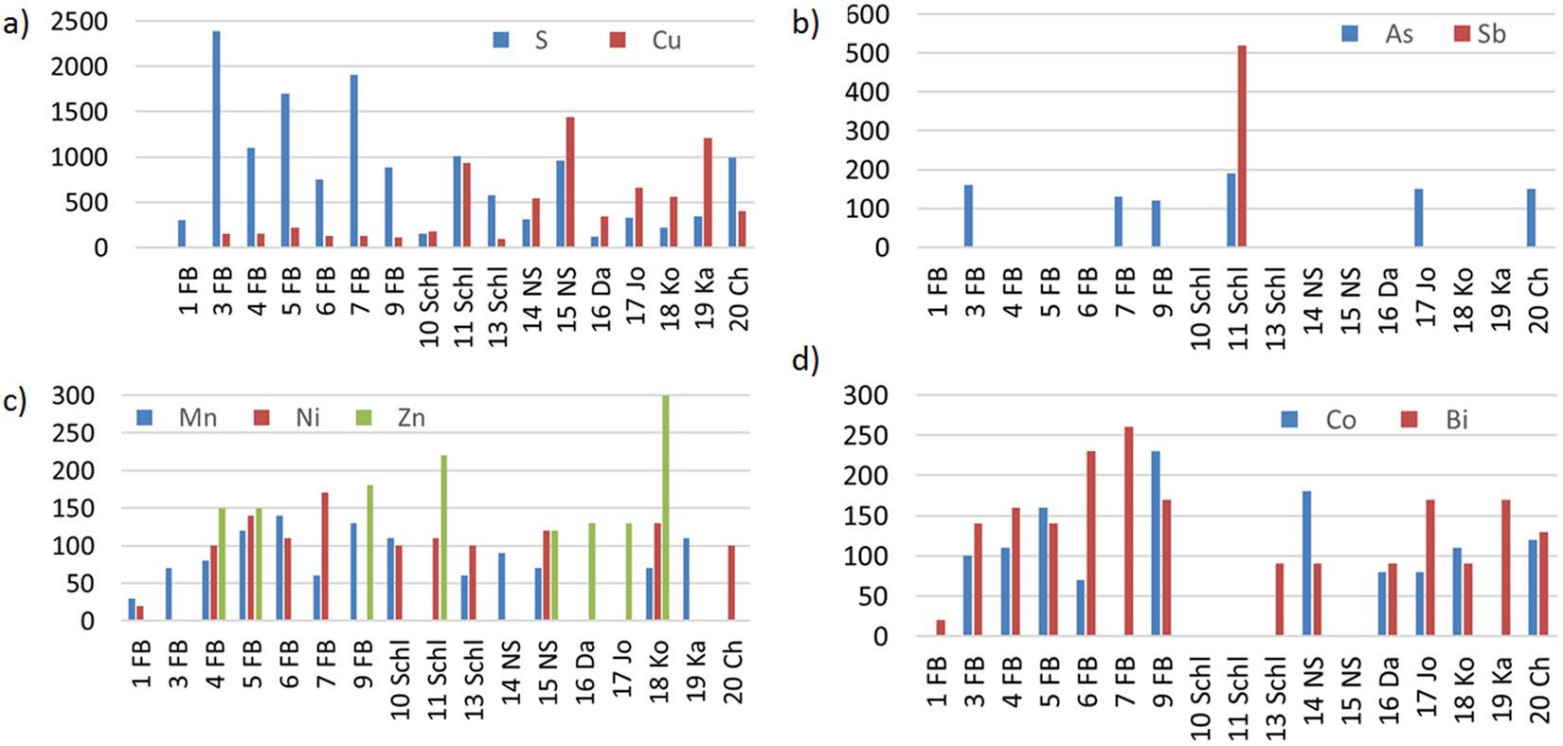
Content of trace elements in the samples [ppm]. (**a**) S and Cu, (**b**) As and Pb, (**c**) Mn, Ni and Zn, (**d**) Co and Bi.

However, various trace elements (<1000 ppm) have been identified which have not been reported previously. The maximum contents found in each specimen have been assigned to the diagrams in Fig. 1. Missing bars indicate that the respective element was either not detected or the element content ranges below the EMPA detection.

In the investigated samples, sulfur (150 to 2390 ppm) and copper (90 to 1440 ppm) are the dominant trace elements (Fig. 1a). Nearly all investigated samples from the Freiberg area (Erzgebirge, Saxony, Germany) are characterized by a considerably high sulfur content above 750 ppm (0.075 wt.-%). It is thus possible that some sulfur is transported from the natural acanthite matrix into the metallic microstructure of the silver curls, as shown in Fig. 2 of the SI.

This might be an indication that the polycrystalline silver curls grew out of the natural sulfide (acanthite) matrix and were not directly deposited from a hydrothermal fluid on the silver sulfide^11,12^. Acanthite crusts on the silver curl surfaces might also entail higher average sulfur contents. In contrast to samples from the other localities, the silver curls from Freiberg show lower copper contents (Fig. 1a). A high copper content was found, for example, in the silver curl from Kasachstan (19 Ka) which might be explained by the fact that the mine in Dscheskastan is operating for copper.

All other analyzed elements, namely As, Mn, Co, Bi, Ni and Zn are only contained in trace amounts below 300 ppm in the studied samples. Locally, their concentration are even lower than the EMPA detection limits (Fig. 1b–d). Sb was discovered in only one specimen from Schlema. Pb has not been found at all in any of the studied samples. Thus, EMPA has revealed compelling evidence for a high purity of the studied samples from different localities in the Erzgebirge and worldwide. To a limited extent, the chemical composition of the samples might also be used as a geochemical indicator for different occurrences (e. g. high Bi content in most of the Freiberg samples).

The structure of silver wires and curls has first been described more than 100 years ago. Mügge^1^, for instance, studied the longitudinally oriented contours on the surface of silver curls which have also been described in the seventies as bundles^13^. Mügge^1^ further suggested that such contours are related to filaments which grew in a kind of liquid and then transformed into a crystalline solid by keeping their original shape. This includes the assumption that the filaments were originally amorphous, and that the curls represent pseudomorphs of silver crystals after the amorphous hair-like silver. He concluded that the form of the curls and wires caused the distortion and apposition of regular crystals and that continuous grain growth converted them into large silver crystals. He also pointed out that all types of transitions exist between the regular large silver crystals and the curly shape. To sum this up, it has already been suggested more than 100 years ago that silver curls are crystalline. However, Mügge^1^ obviously assumed that each of the longitudinal contours represent individual large crystals.

The same conclusion was drawn in the late twenties by Schenck^2^ who found by pioneering radiographic procedures that naturally grown silver crystals are not preferably oriented along the wire axis. In contrast, an artificially grown wire seems to exhibit a preferred orientation along the wire axis which vanishes after a heat treatment at 900 °C.

The nucleation of silver curls and their longitudinal contours have been discussed in the late seventies^13^ as a result of the surface roughness of the substrate, in this case acanthite.

**Figure 2:**
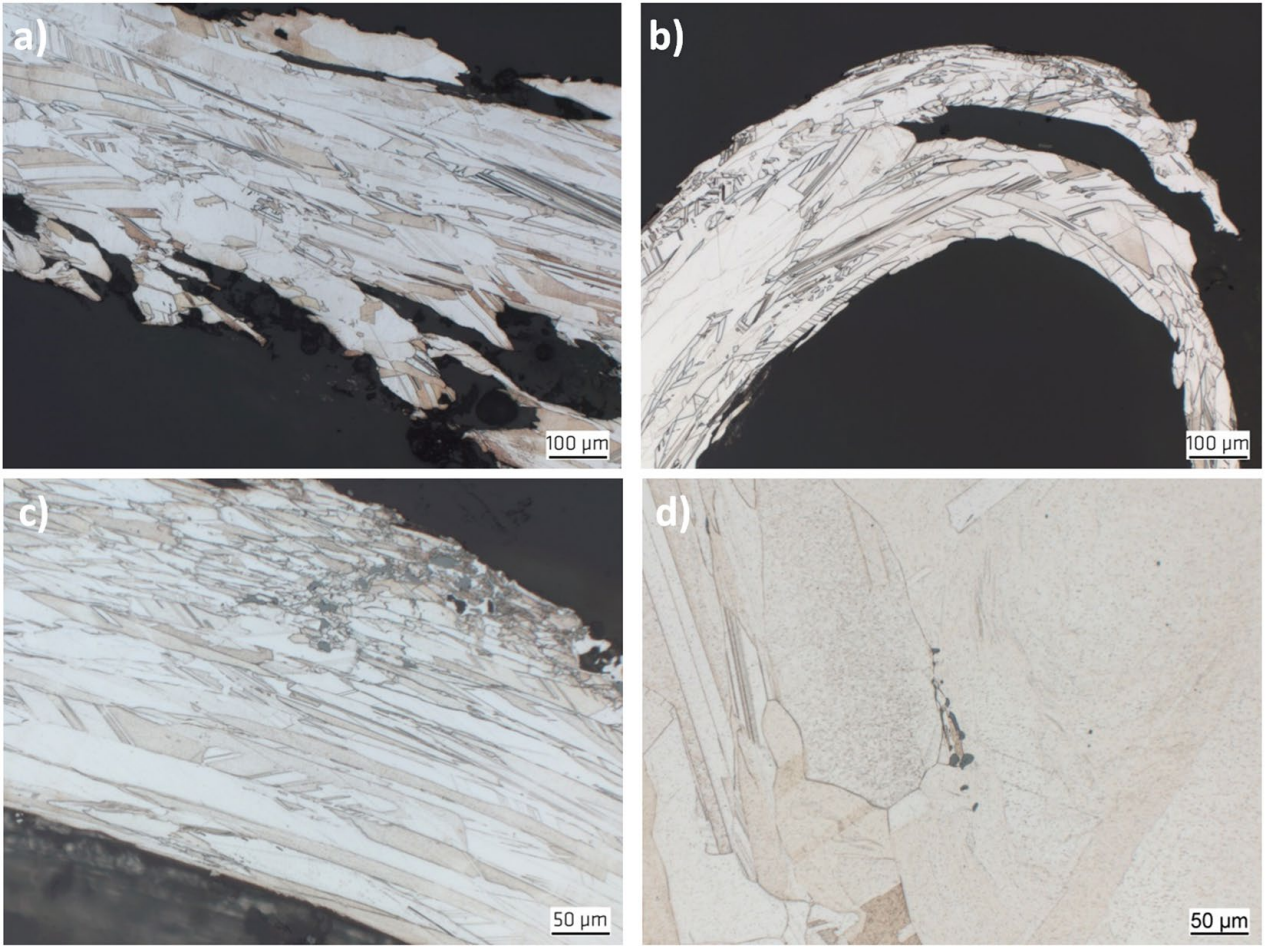
Typical microstructures of natural wire silver specimens. The images show longitudinal section of (**a**) the center section of the silver curl from Sample 15 NS, (**b**) the top region of a silver curl from Sample 18 Ko, (**c**) a silver curl from Sample 10 Schl with inclusions of acanthite and (**d**) a silver curl from Sample 19 Ka with inclusions of stellerite.

Optical microscopy revealed evidence for a polycrystalline metallic microstructure for all studied samples. Some exemplary micrographs are shown in Fig. 2. In contrast to the observations of Mügge^1^ and Schenck^2^, but consistent to later findings by Ohachi and Taniguchi^13^, the longitudinal contours are further subdivided into smaller crystals and do not represent single-crystalline volumes, i.e. grains. After comparison of all samples it turned out that their microstructure tends to be very similar. This indicates a similar generation process for all investigated natural silver curls. Especially in thicker curls, the discovered microstructure exhibits arrangement of twinned grains which is fairly common for melt-grown face-centered cubic (fcc) metals, like copper, nickel alloys or austenitic stainless steels.

The average grain size in perpendicular to the wire axes ranges at around 50 µm. Only two specimens exhibit larger grain dimensions.

However, in all specimens the grains are significantly elongated parallel to the wire axis (Fig. 2a,c). Frequently, grains appear more than ten times longer than their diameter, i.e., their length-to-width ratio reaches values above 10. The average grain size of all specimens in the longitudinal direction ranges at about 500 µm and grain lengths of even more than 1 mm have been observed. Typically, such extremely elongated grains appear more frequently towards the top end of the wires (Fig. 2b).

At the roots of the wires, the grains appear generally smaller and more equiaxed in size in the transverse direction. This indicates a homogeneous nucleation of the polycrystals. In some specimens, also mineral inclusions like Ag_2_S have been observed (Fig. 2c,d).

**Figure 3:**
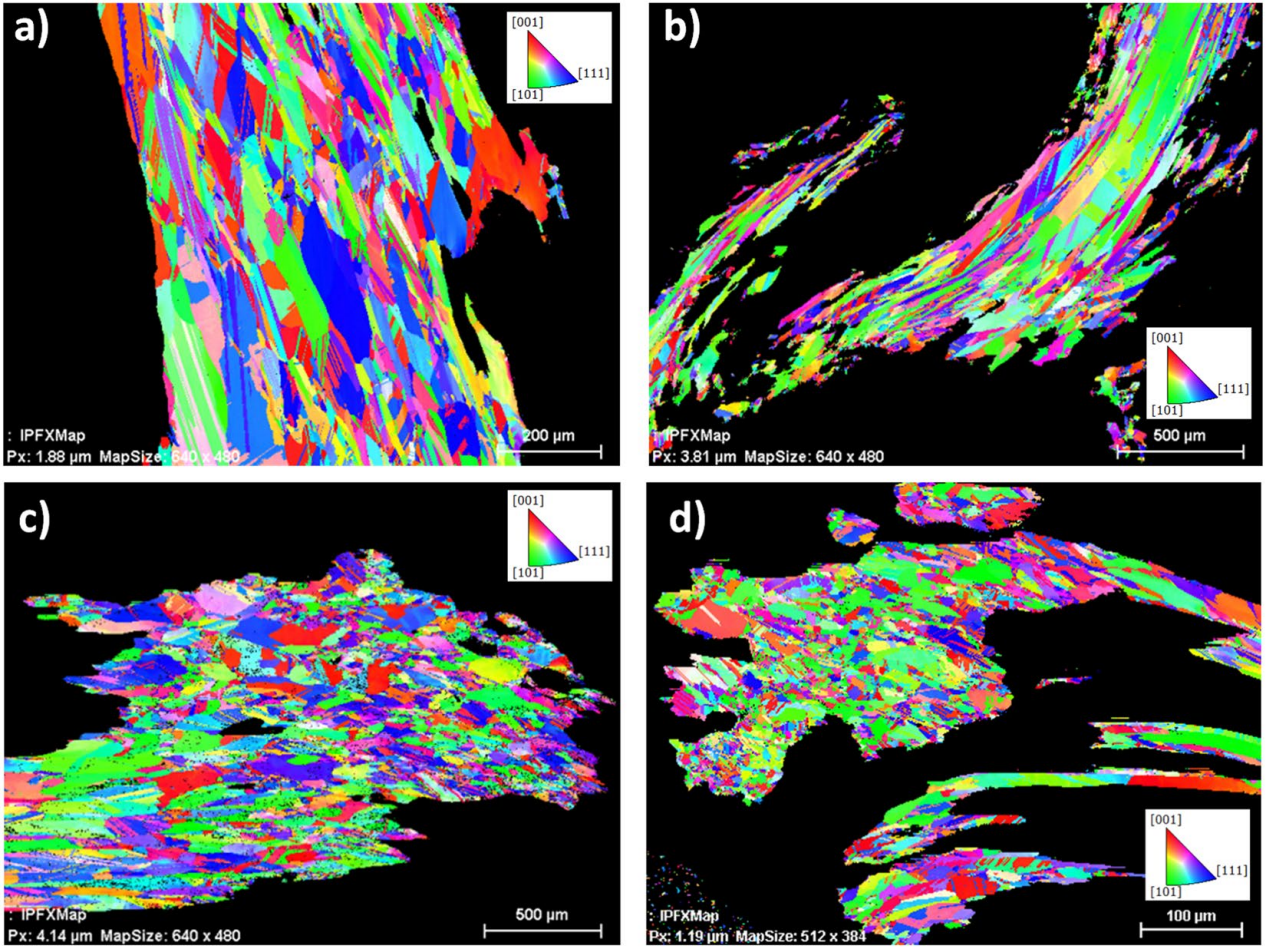
Typical EBDS IPF mappings of (**a**) a longitudinal cross-section in the center of the wire Sample 14 NS, (**b**) a longitudinal cross-section in the tip of the wire Sample 14 NS, (**c**) a transverse cross-section in the center of the wire Sample 15 NS and (**d**) a transverse cross-section at the root of the Sample 20 Ch.

The optical micrographs in Fig. 2 already suggest significant twinning of grains. This can be proven quantitatively by orientation maps using EBSD. Displayed by an Inverse Pol Figure (IPF) coloring^14^, twins are indicated by two different colors which alternate along straight lines, if multiple twins of the same crystallographic character occur. Figure 3 represents four orientation maps collected in different areas at different magnifications within curls of different samples. Because of the overview character of the maps the existing twinning is not that obvious in Fig. 3a. However, a closer look on the maps in the Fig. 3b–d also shows a considerable amount of twins. Especially when twin boundaries are not straight anymore, a deformation cannot be denied. However, curling does not automatically mean bending of the internal microstructure (Fig. 2b). The microstructure displays a very strong curling whereas the visible twin boundaries practically do not show any bending.

Figure 3a,b represent longitudinal sections of curled silver wires from the former uranium mines at Niederschlema-Silberbachsthal, Saxony, Germany. The elongated shape of the grains is even more pronounced in Fig. 3b which has been taken from the tip of a different curl from the same specimen. No significant changes of grain size and shape is expressed in the EBSD map in Fig. 3a. In contrast, Fig. 3b, reflects clearly elongated grains. This increased form factor indicates faster growth. In the region of the curl tip compared to the center or the root of a curl. The EBSD maps shown in the Fig. 3c,d suggest a more equiaxed grain dimension, however, because of the complex shape and alignment, the sections prepared are quite accidentally oriented. Equiaxed grains commonly indicate a preparation perpendicular to the growth direction. Thus, the main part of the map in Fig. 3d represents the root of a thin wire.

It starts with a very pronounced fine-grained microstructure and leads to respective longitudinal growth of the crystals, resembling homogeneous nucleation in melt-grown microstructures.

Generally, significant twinning has been observed in all samples, independent from the investigated region. Straight boundaries, less narrow twinning planes and partially, terminations inside grains^15–17^ are typical features for annealing and growth twins and become quite obvious in Fig. 3a,b. Many of the twins in the natural silver curls might thus be attributed to growth effects and accidents on the <111> -planes.

However, twinning and cross-twinning in metallic microstructures can extensively mediate the grain growth^17,18^. Although this is attributed to grain growth at elevated temperatures, it might perhaps have an influence on the preferential growth of the grains in the longitudinal direction.

**Figure 4:**
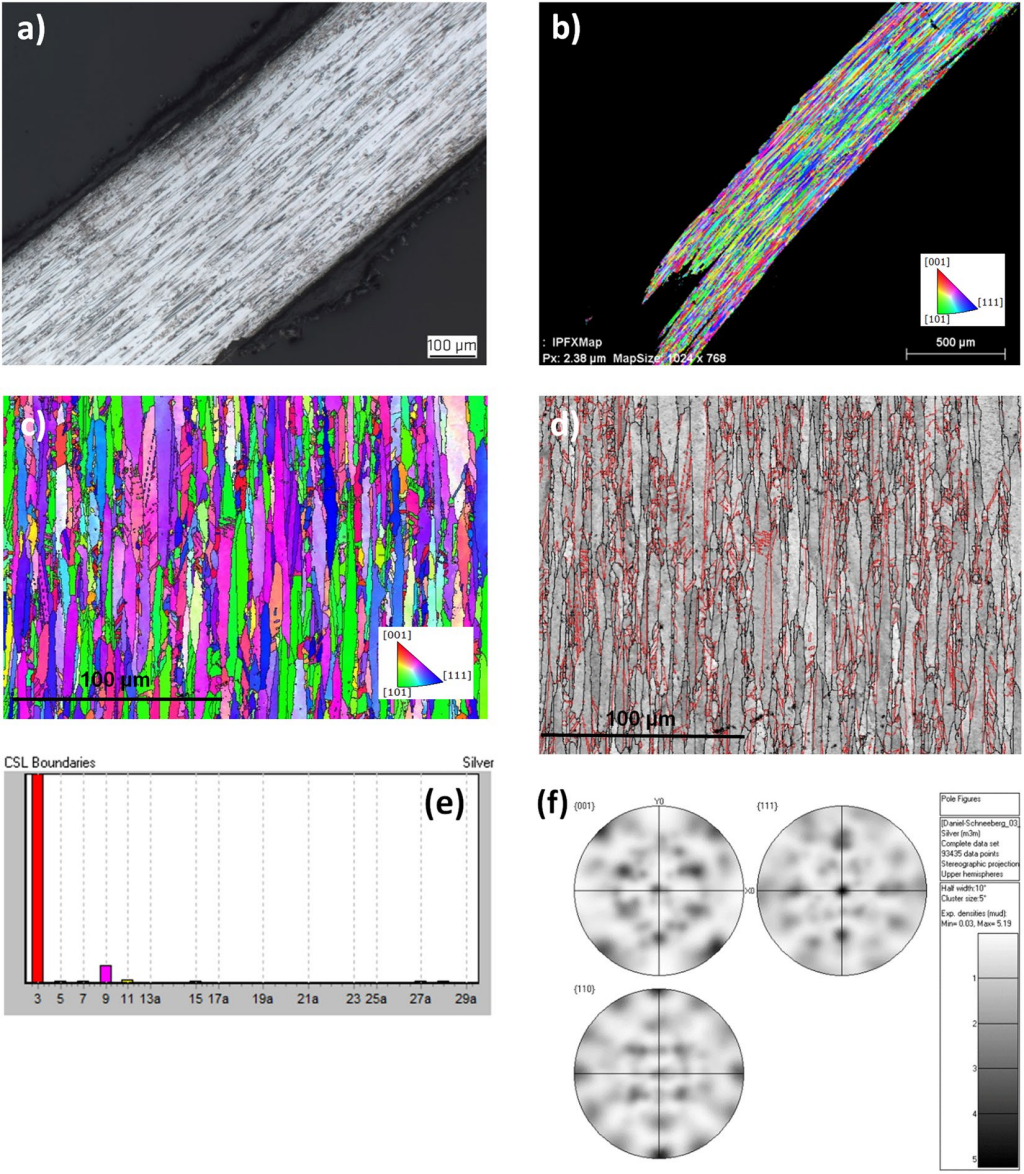
Structural and microstructural characterization of Sample 16 DA by (**a**) light-microscopy of a longitudinal section through a wire, (**b**) IPF color coded orientation map of a region close to the microstructure shown in Fig. 5a, (**c**) EBSD map coloring the vertical direction (parallel grain axes) in terms of crystallographic indexing (in case of a fiber texture all grains would have a similar color), (**d**) Band contrast map (EBSD) overlaid by high-angle (black) and twin boundaries (red) with dominant boundaries as high angle boundaries whereas internal boundaries are preferable twins, (**e**) Distribution of twin boundaries, abundant Σ3 twin boundaries, only small amounts of multiple twin boundaries Σ9 and Σ27 and (**f**) Pole figures displaying at least an orthorhombic symmetry, possibly also a cubic symmetry, if the different weight of texture components is considered.

For a more detailed investigation of the microstructure formed in hair-like natural silver, thin wires of the Specimen Da 16 have been selected. This should help to understand the different microstructural features compared to thicker curls. As shown by the light microscopy image in Fig. 4a as well as by the comparable orientation map in Fig. 4b, the selected hair-like silver is dominated by the expected elongated grains. Grains with a length-to-width ratio above 20 are typical for this specimen. An orientation map collected at higher magnification (Fig. 4c) reveals similar abundant twinning as in the thicker curl-like natural silver wires. The wire in Fig. 4a has been aligned in a way that in case of a fiber texture the vertical reference direction (IPF-Y) would be crystallographically preferred for all grains. If there would be a fiber texture, all grains should be colored equally. The color variation in the Fig. 4b,c, however, shows that the long axes of all grains are differently indexed, i.e. crystallographically not equivalent. Interfaces between elongated grains are defined by high-angle boundaries (Fig. 4d). It also shows that the dominating high-angle boundaries are no twin boundaries (red) or any other coincident lattice boundary. Beside twin boundaries, Σ3, only a small amount of multiple twin boundaries Σ9 and Σ27 occur (Fig. 4e). Figure 4c suggests a higher amount of green, blue and violet colored grains. Analyzing possible texture components, a subset of grains prefer as elongated axis <110> or <111> . All violet colored grains, however, have their <111> mainly perpendicular to the elongated grain axis. Other maps confirm the impression that the resulting pole figure (Fig. 4f) mainly represents an orthorhombic system possibly inherited from a former single crystal, most likely argentite (orthorhombic, pseudocubic). Since it transforms into different orientation variants of acanthite, the argentite symmetry is still visible in the acanthite as long as the silver wire covers the former area of a single acanthite only. If two or more argentites contribute to a silver curl, the inherited orientation relationships are not clearly recognizable anymore.

As mentioned before, as driving force of the longitudinal growth of whiskers screw dislocations have been proposed^17^. In contrast, Ohashi and Taniguchi^13^ imprecisely naming their investigated wire silver samples whiskers, proposed that the twins are a result of supersaturation. However, the mechanism of single-crystal whisker growth and polycrystalline wire silver growth might be similar, since in both cases the growth happens obviously via the basal plane of the wire/whisker. Whiskers normally grow on the same material, e. g. Ag on Ag. Whiskers are single crystals, but they do not have any recognizable orientation relationships to the substrate. In contrast, silver wires or curls require additional phase transformation from a silver bearing mineral to silver. Moreover, they consist of thousands of grains which do not have an inherent geometrical orientation relationship like the well-known coincident site lattices. On the other hand, their pole figures represent an inherent symmetry mmm which only fits to a transformation relationship. Since acanthite is monoclinic, only the high-temperature modification argentite explains the visible symmetry.

**Figure 5:**
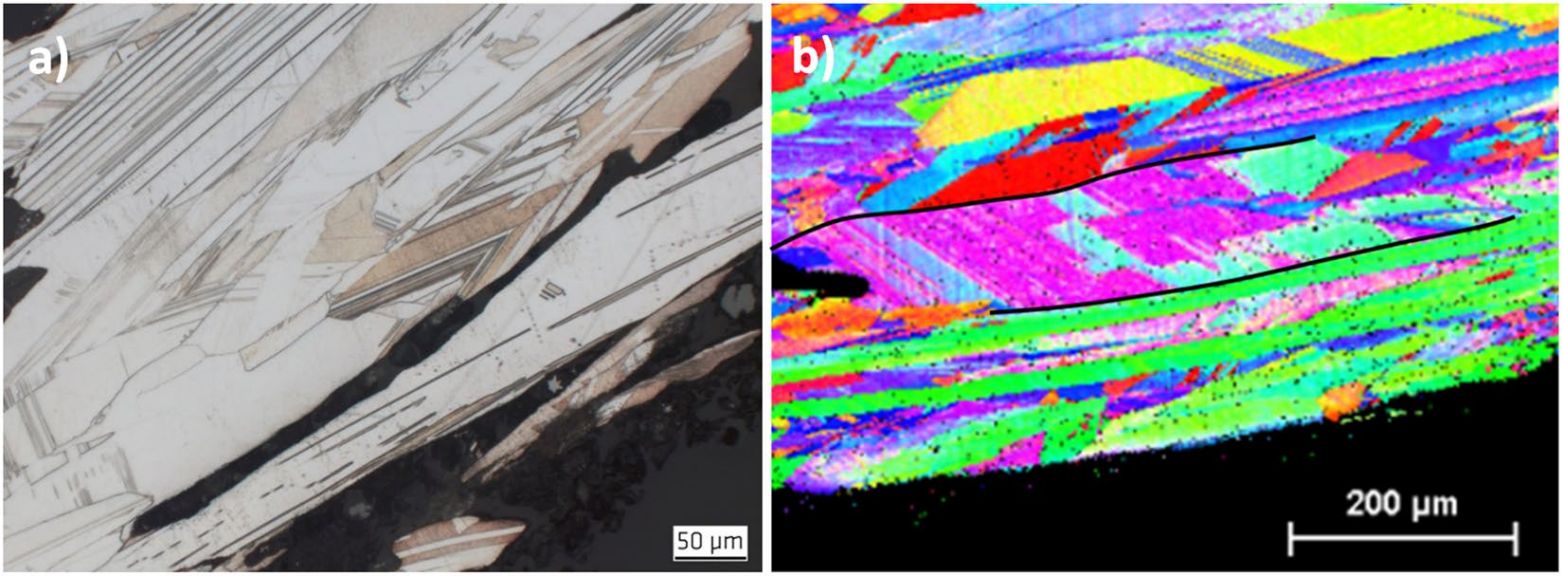
Different twinning types in the natural silver fcc polycrystals. (**a**) Region with extreme and predominantly deformation twinning in the Specimen 15 NS and (**b**) IPF from EBSD of the elongated grains in the Specimen 10 Schl, exhibiting both, growth twins as well as deformation twinning and 45° slipping.

However, all polycrystalline fcc metals tend also to exhibit deformation twinning. As typical features, it has also been observed in some regions that the slim twins cover the whole grain size and exhibit end tips (Fig. 5a). As also typical for deformation twinned regions, slipping with its typical patterns of tightly aligned parallel lines in the typical 45° direction of the cubic system of the silver crystals has been observed (Fig. 5b). Deformation related slipping and twinning occurred generally in more curved and even curled top regions. It can thus not be excluded that at least some of the specimens have been artificially treated, i.e. they might have been curved mechanically or even sloped afterwards for improvement of their appearance.

**Figure 6:**
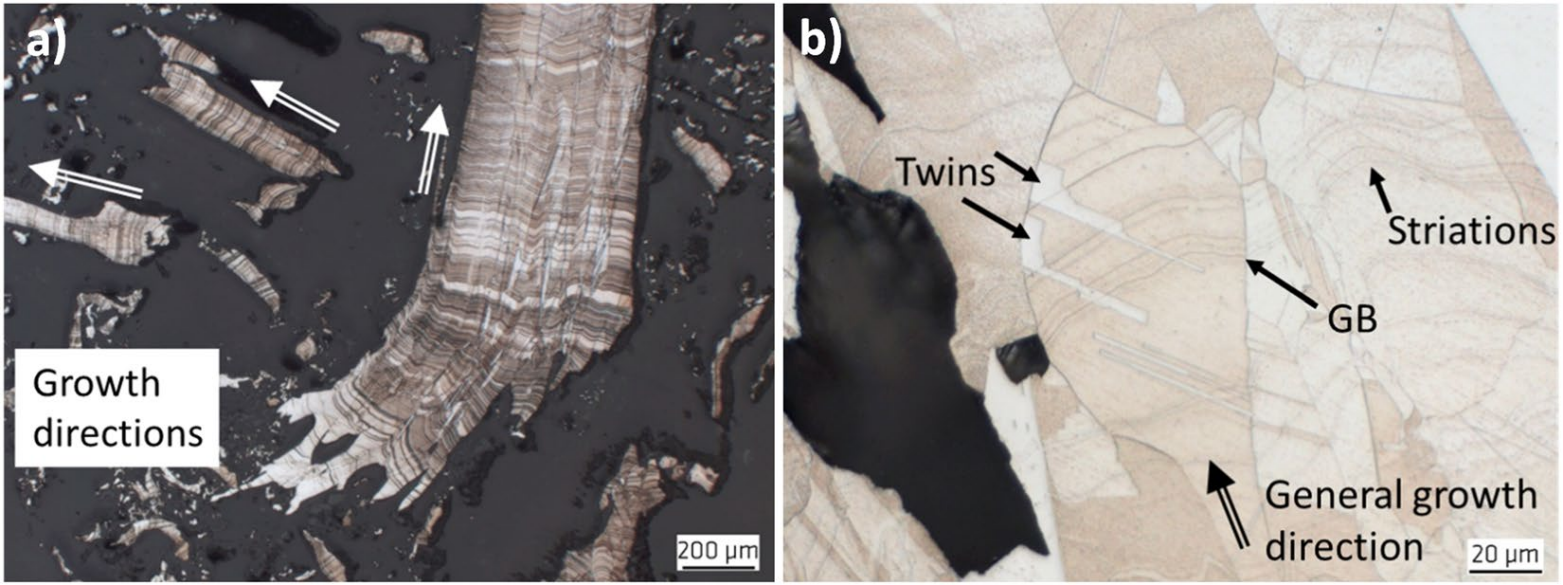
Curved striations in the Sample 17 Jo. (**a**) Overview of several silver curls growing in several directions like a nest on the sample and (**b**) Close-up of a cross section of the same curl with striations altering curvature at grain boundaries (GB).

In contrast to the longitudinal contours of the silver curls described above, on the cross sections of Specimen 17 Jo, parallel striation has been observed that is oriented transverse to the growth direction, as shown by the cross sections in Fig. 6. Such lines are significantly curved and have evidently been observed on the outer surface of the wires as well. As shown in Fig. 6b, these striations obviously change their curvature at grain boundaries, but not at twins. At a first glance, these lines might be interpreted as segregation curves. But, such striations seem to be formed independently from any grain boundaries or twinning (Fig. 6b) and might be taken as growth lines and thus, represent an indication for oriented growth. Together with the typical polycrystalline microstructure, this might indicate that the mechanism during growth of the natural silver curls is similar to melt growth^13^.

**Figure 7:**
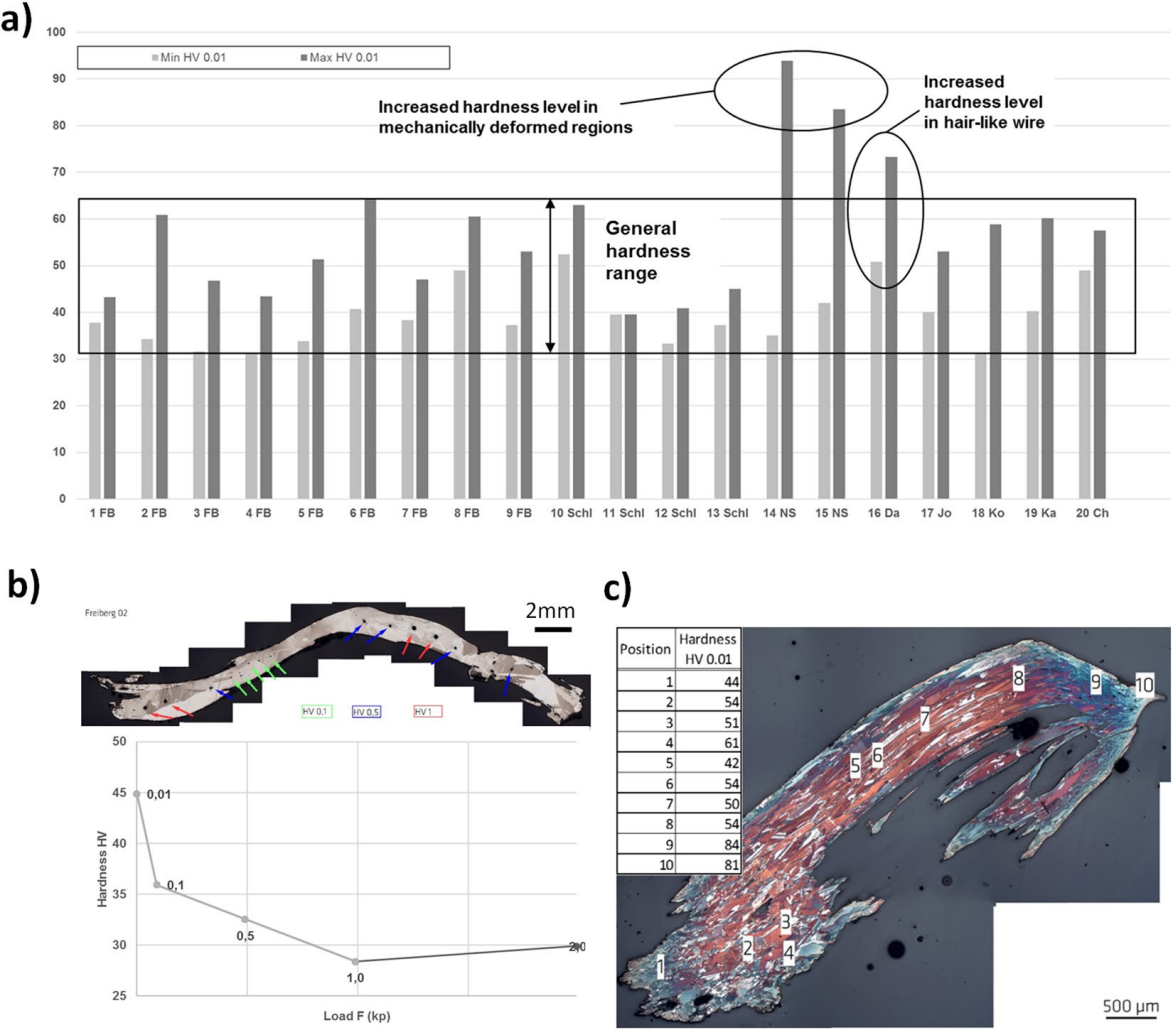
Vickers-hardness of the investigated natural silver specimens. (**a**) Minimum and maximum hardness values HV 0.01. (**b**) Different values for HV 0.01 to HV 2 in the Specimen 2 FB and (**c**) Hardness values of the Specimen 15 NS increasing significantly in cold-deformed regions.

Vicker’s hardness measurements have been carried out for the first time and on all 20 wire silver specimens. Correlation of the Mohs’s scale of hardness with the Vicker’s hardness scale^18–20^ is described in the supplementary information. For evaluation of hardness changes dependent on specific microstructural features, HV 0.01 has been applied to the thin wire silver specimens. The respective minimum and maximum values have been assigned to the diagram in Fig. 7a. Figure 7b shows the locations of the indentation points along specimen 2 FB as an example. As can be drawn from the bar chart, the values of the investigated microstructures range between 35 and 65 HV 0.01 (Fig. 7a).

The rough conversion of the frequently provided Mohs’s hardness value for natural silver of 2.7 into the Vicker’s hardness as shown in the supplementary information provides already a higher value of 76.6 HV. In addition, the Vickers hardness is rapidly increasing for decreasing indentation loads. Both together indicates that micro-hardness of HV 1 or HV 2 applied normally to polycrystalline fcc metallic microstructures will range for natural silver curls at much lower values than it might be generally assumed by simple conversion of the Mohs into the Vickers hardness.

Significantly higher maxima of the hardness values over 80 HV have been obtained for the specimen 14 NS and 15 NS (Fig. 7c) which were obtained in significantly distorted micro-structures at the root of this curl where it has been mechanically cut off from the matrix. Exceptionally high hardness levels thus represent an indication for mechanical deformation and artificially introduced cold work into natural silver curls. An increased hardness range has also been obtained for the natural silver wire 16 Da that can also be easier mechanically deformed, due to the thin hair-like structure.

## Conclusions

From the present investigation of 20 natural silver wires from various global occurrences the following conclusions can be drawn:

1. The chemical composition shows a quite high purity of all natural silver curls with above 99.7 wt.-% Ag. As trace elements, S, Cu, Co, Bi, Mn, Ni and Zn have been detected in nearly all specimens. As appears more scarcely, and Sb has only been found in two of 17 analyzed curls. No Pb has been found any of the studied samples.
2. Natural silver wires in hair- and curl-like structures exhibit a face-centered cubic (fcc) polycrystalline microstructure similar to melt-grown metallic microstructures, irrespective to the completely different geochemical nucleation and growth process. The grains are mostly equiaxed in the transverse direction at a size of around 50 µm, but are extremely larger in the longitudinal direction of the curls with a length-to-width ratio of 10 to 20 that might become even larger in hair-like silver wires.
3. All grains of the investigated natural silver curls exhibit significant growth twinning, as indicated by straight boundaries, less narrow twinning planes, terminations inside grains and no passing of grain boundaries. Such twinning occurs similarly in more curl-like as well as in thin hair-like silver wires. The difference between twin and grain boundaries provides clear evidence for the polycrystalline microstructure hair-like silver wires, as compared to single crystal whiskers. Differences and similarities of the microstructure and growth mechanisms between silver whiskers and natural wire silver needs be investigated further in detail.
  Extensive deformation twinning has only been observed in heavily cold-worked regions of the natural silver wires and might represent an indication for later mechanical treatment to improve the appearance as collectibles.
4. As most suitable procedure to cover also microstructural influences on the hardness in the partly thin sections, determination of the Vickers hardness HV 0.01 has been identified. The maximum and minimum hardness values range at much lower levels when compared with those derived from simple conversion of the Mohs into Vickers Hardness. In contrast, significantly increased hardness values have been identified in cold-worked regions of the silver wire microstructures, which also might provide indications for later mechanical treatment of the natural grown specimens.

## Methods

Samples have been taken from 20 silver wire specimens originating from various localities. The origin of the samples is provided in Table 1 in the supplementary information. Cross sections have been prepared at BAM, preferentially along the longitudinal direction of the wires. Reflected light microscopy has been carried out in the grinded and polished state, but also in the etched condition. A 20 vol.-% alcoholic nitric acid turned out as best etchant to elucidate the various microstructural features of the various silver wires.

Trace element concentrations have been measured by Electron Microprobe Analysis (EPMA). The concentrations of S, As, Cu, Pb, Zn, Mn, Cu, Co, Ni, Sb in silver were determined in 17 polished sections at GFZ Potsdam using a JEOL.JXA-8500F Hyperprobe equipped with a field emission gun and five wavelength-dispersive spectrometers. Detection limits of 0 ppm for Pb, 97 ppm for As, 76 ppm for Sb, 87 ppm for Bi, 64 ppm for Co, 86 ppm for Ni, 91 ppm for Cu, 116 ppm for Zn and 22 ppm for S were obtained with a 1 µm diameter beam at 20 KV and 40 nA. Counting times were 100 s on peak for S, As, Pb, Sb and Bi, 80 s on peak for Co, Ni, Cu, Zn and 60 s on peak for Mn, with additional half count rate for background measurements on both sides of the peak. Calibration was done using standard material.

In total, 162 analyses were considered for this study.

For subsequent electron back scattering diffraction (EBSD) analyses at BAM, all samples have been subjected above metallographic standard preparation followed by a 3 min surface finishing by Masterprep (Buehler). Despite a very careful preparation, this standard procedure implements a practically inconspicuous surface deformation which has been removed by a multiple Ar-ion etching (PECS, Gatan), until all intracrystalline orientation variations disappeared. The orientation maps have been generated using a Bruker EBSD system attached on a field-emission SEM LEO1530 VP (ZEISS). For all analyses, an acceleration voltage of 20 kV and a beam current of about 7 nA were used. For reduction of charging a chamber pressure of 13 Pa was chosen. The collected EBSD patterns were binned down on-chip to a size of 160 × 115 pixels.

The micro-hardness of the various specimens has been analyzed by a common Vickers test apparatus at BAM. Since the silver wires were partly very thin, the smallest load of 0.01 kp (0.0981 N) has been applied and HV 0.01 has been determined. Measurements of up to HV 2 with a load of up to 19.6 N (20 kp) at a curl with a larger diameter revealed nearly the same hardness levels of 28.31 and 29.92 for HV 1 and HV 2, respectively, in contrast to the measured value of 44.89 for the hardness HV 0.01. To derive a relation to the micro-hardness HV 1 and HV 2, commonly used for measurements of softer metallic microstructures, the hardness HV 0.01 applied here for the small natural silver specimens can be divided by 1.5. Note that such conversion factors are dependent on the type of the investigated material and microstructure, respectively, and cannot be transferred to any other type of metallic material.

## Electronic supplementary material

Supplementary Information
