# Supplementary material for: Microstructural Insights into Natural Silver Wires
Source: Sci Rep. 2018 Jun 13;8:9053. doi: 10.1038/s41598-018-27159-w (PMC5998055; doi:10.1038/s41598-018-27159-w)
Supplement: Supplementary file 1 — Supplementary Information [file 41598_2018_27159_MOESM1_ESM.pdf]

MICROSTRUCTURAL INSIGHT INTO NATURAL SILVER WIRES

SUPPLEMENTARY INFORMATION

Th. Boellinghaus[1,]*, V. Lüders[2], G. Nolze[1]

[1]BAM – Federal Institute for Materials Research and Testing,

Unter den Eichen 87, D-12205 Berlin, Germany

[2]GFZ – German Research Centre for Geosciences,

Telegrafenberg, D-14473 Potsdam, Germany

*Corresponding authors email: thomas.boellinghaus@bam.de

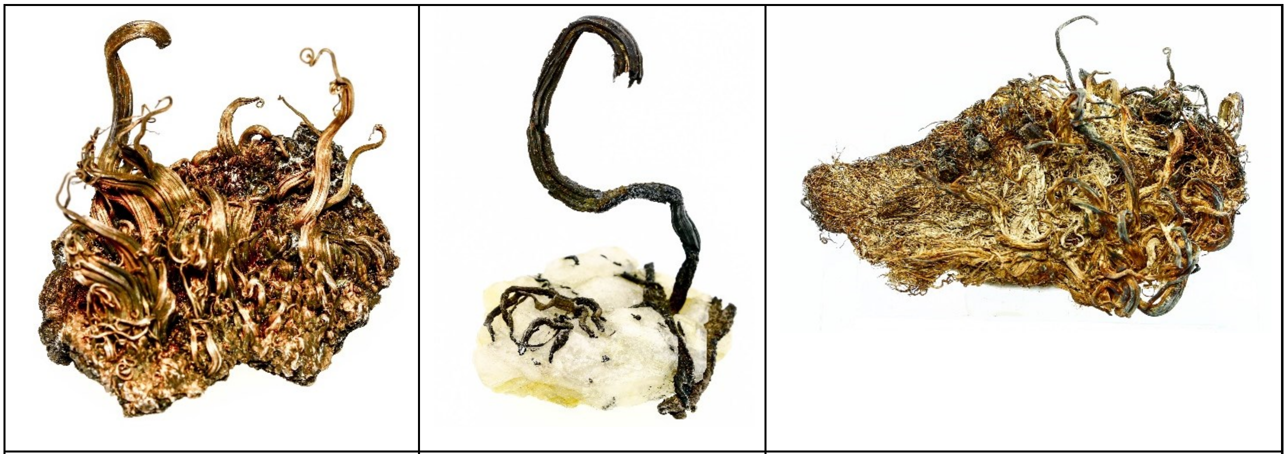

a) Lager and smaller curls | b) Single curl | c) Hair like structure

Figure 1: Natural wire silver specimens from Schlema, Erzgebirge, Germany

Table 1: Overview of samples from wire silver specimens from various localities (bold bars: cm, small bars: mm)

Spec.	Locality of origin	Macrograph	Spec.	Locality of origin and features	Macrograph
1 FB	Freiberg, Erzgebirge, Germany	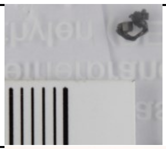	11 Schl	Schlema, Erzgebirge, Germany	
2 FB	Freiberg, Erzgebirge, Germany	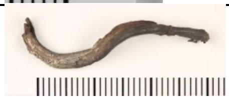	12 Schl	Schlema, Erzgebirge, Germany	
3 FB	Freiberg, Erzgebirge, Germany	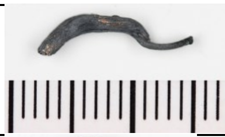	13 Schl	Schlema, Erzgebirge, Germany	
4 FB	Freiberg, Erzgebirge, Germany	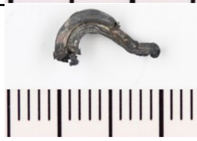	14 NS	Niederschlema, Erzgebirge, Germany	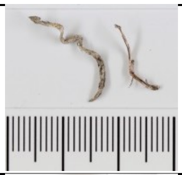
5 FB	Freiberg, Erzgebirge, Germany	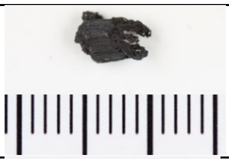	15 NS	Niederschlema, Erzgebirge, Germany	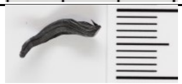
6 FB	Freiberg, Erzgebirge, Germany	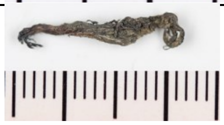	16 Da	Daniel Mine, Schneeberg, Germany	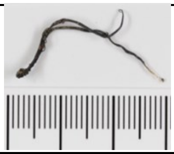
7 FB	Freiberg, Erzgebirge, Germany	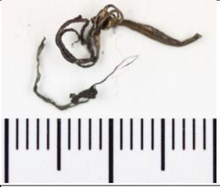	17 Jo	Jachymov, Czech Republic	
8 FB	Freiberg, Erzgebirge, Germany	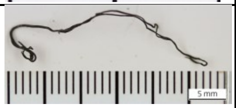	18 Ko	Kongsberg, Norway	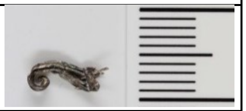
9 FB	Freiberg, Erzgebirge, Germany	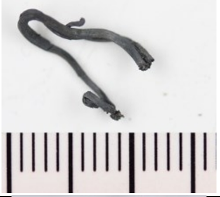	19 Ka	Dscheskasgan, Kazakhstan	
10 Schl	Schlema, Alberoda, Erzgebirge, Germany	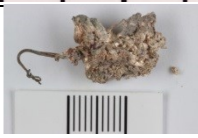	20 Ch	Wutong Mine, Liubao, Wuzhou Prefectrure, Guanzi Zhuang Autonomous Region, China	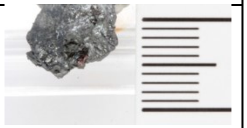

Formation of natural silver wires

Native silver enrichments are typically found in oxidation zones of silver and/or sulfide deposits where groundwater and/or descending solutions infiltrated into the upper parts of the deposits.[1] Here, silver wires and curls are commonly grown on Ag-bearing sulfides such as acanthite (Ag_2S), but may also occur in open fractures or are overgrown by gangue minerals such as carbonates. At the moment, no statement can be made about the formation temperatures of natural silver wires. Although natural silver crystals and dendrites can deposit from hydrothermal fluids, native silver wires and curls may be the product of low temperature (electrochemically triggered) alteration processes of Ag-bearing sulfides.[2] Supergene silver formation due to oxidation of Ag-bearing sulfides is schematically shown in Figure 2.

During alteration of acanthite or Ag-bearing sulfides by oxidizing fluids sulfur is released as SO_2 (Equation (1) in Figure 2) and may get further oxidized to SO_4^{2-}. In the presence of Ba^{2+} barite may precipitate.

Silver atoms (Equation (2) in Figure 2) remain on the sulfide´s surface and can accumulate to a nucleus of the later silver wire. Part of the uncharged Ag° atoms at the surface can be ionized releasing electrons to the substrate and silver ions (Ag^+) to the aqueous phase.

Such Ag^+ ions are attracted by the negative electric field (δ^-) of the growing silver wire. The electric field strength is highest at the top of the growing silver wire and thus linear crystal growth is favored. This process can continue until the host sulfide is completely consumed or the system dries up.

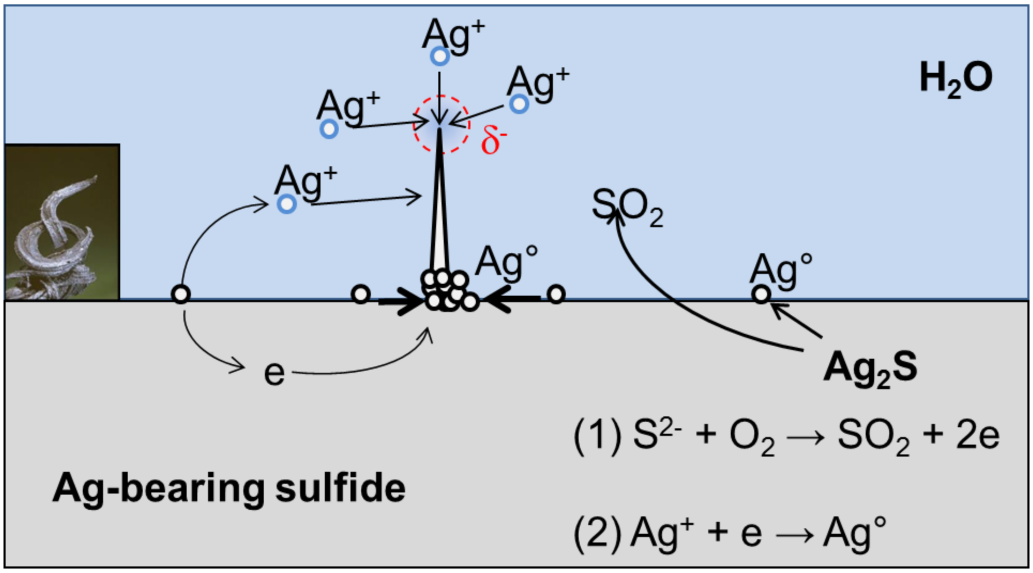

Figure 2: Growth of natural silver wires by alteration of Ag-bearing sulfide

Hardness measurement for silver

Up to now, the hardness of natural silver specimens is mostly very roughly evaluated by the Mohs Hardness (MH) which is not much precise as a scratch method. Generally, values between MH = 2.5 and MH = 3 have been reported. The Mohs scale between 1 and 10 is normally applied to minerals. Anyway, such differentiation is much too rough for metallic microstructures which are usually evaluated by the Vickers Hardness (HV), based on indentation measurements. Conversion equations or charts between the Vickers and Mohs hardness values do nearly not exist, except the rough table published by Tayler[3] and the simple conversion equation given by Herrmann et al. [4]:

$$\log HV = -0.90394 + 1.56269MH - 0.22907MH^2 + 0.01213MH^3$$

Applying the conversion equation to the reported MH results in a Vickers hardness range roughly between HV 50 and HV 100. However, the equation does not consider that the respective VH value is strongly dependent on the applied load on the indenter, ranging from 0.01 kp = 0.0981 N in the micro hardness range providing information about the hardness in specific grains to 10 kp = 98.1 N in the macro scale providing an average information of a specific microstructure over several grains. The values in the micro-hardness range below HV 1 show a very steep, nearly exponential, ascent towards higher values, while the ascent is much smaller and normally linear towards higher applied Vickers indentation loads between HV 1 and HV 10 in the so-called macro-hardness range.

HV values are thus only valid with the applied load mentioned as an extension, like HV 0.01 or HV 10. The conversion table provided by Tayler is valid for a Vickers indentation load of 0.05 kp, i. e., it correlates to HV 0.05. Under the consideration of increasing hardness values for decreasing indentation loads and the range of values roughly calculated by conversion of the Mohs´s hardness[3,4], the frequently reported Mohs´s hardness value for natural silver of 2.7 giving 76.6 HV is already significantly higher than the measured Vickers hardness values within this study. For instance, assuming a linear relationship between Vickers and Mohs´s hardness, this would result for the above mentioned Mohs´s hardness in a Vickers hardness range between 82.5 and 105 HV 0.05.

References

1 Pohl, W. L. *Economic geology: principles and practice*. (Wiley - Blackwell, 2011).

2 Wagner, C. Investigations on silver sulfide. *The Journal of chemical physics* **21**, 1819-1827 (1953).

3 Taylor, E. W. Correlation of the Mohs's scale of hardness with the Vickers's hardness numbers. *Mineralogical Magazine* **28**, 718-721 (1949).

4 Herrmann, K., Kompatscher, M., Polzin, T., Ullner, C. & Wehrstedt, A. *Härteprüfung an Metallen und Kunststoffen: Grundlagen und Überblick zu modernen Verfahren; mit 66 Tabellen*. (expert verlag, 2007).
